# High Quality Bergamot Oil from Greece: Chemical Analysis Using Chiral Gas Chromatography and Larvicidal Activity against the West Nile Virus Vector

**DOI:** 10.3390/molecules14020839

**Published:** 2009-02-18

**Authors:** Melliou Eleni, Michaelakis Antonios, Koliopoulos George, Skaltsounis Alexios-Leandros, Magiatis Prokopios

**Affiliations:** 1 Department of Pharmacognosy and Natural Products Chemistry, Faculty of Pharmacy, University of Athens, Panepistimiopolis-Zografou, Athens 15771, Greece; E-mails: emelliou@pharm.uoa.gr (M.E.), skaltsounis@pharm.uoa.gr (S.A-L.); 2 Laboratory of Agricultural Entomology, Department of Entomology and Agricultural Zoology, Benaki Phytopathological Institute, 8 S. Delta Str. 14561 Kifissia Athens, Greece; E-mail: a.michaelakis@bpi.gr (M.A.); 3 Laboratory of Insecticides of Public Health Importance, Benaki Phytopathological Institute, 8 S. Delta str. 14561 Kifissia Athens, Greece; E-mail: g.koliopoulos@bpi.gr (K.G.)

**Keywords:** Bergamot, *Citrus bergamia*, Essential oil, GC-MS, Mosquito, *Culex pipiens*

## Abstract

The essential oils contained in the rind of the fruit and the leaves of bergamot from Greece (*Citrus aurantium subsp. bergamia*) were studied. The bergamot trees in question were cultivated on Kefalonia Island. The plant material (leaves and fruits in different stages of maturity) was collected between December and March for a two year period. The rind of the fruit was separated manually and the essential oil was obtained either by cold pressing or by hydrodistillation. The maximum yield calculated on a wet weight of fresh rinds basis was 1.8%. The essential oils were first analyzed by GC-MS with a DB-5 column and then with a β-Dex™ enantiomeric column. The main constituent of the cold pressed essential oil of the rind was (–)-linalyl acetate with optical purity >99.9%. Other important constituents were (–)-linalool, (+)-limonene and γ-terpinene. The best value of linalool/linalyl acetate ratio was 0.38 and the maximum sum of linalool+linalyl acetate was found to be 55.8%. The larvacidal activities of the obtained essential oils and the compounds (±)-linalyl acetate, (±)-linalool and (–)-linalool were evaluated against larvae of the mosquito species *Culex pipiens* (Diptera: Culicidae), the West Nile virus vector, under laboratory conditions. The cold pressed essential oil showed an LC_50_ value of 58 mg/L, while the LC_50_ value of the corresponding essential oil obtained by hydrostillation was 106 mg/L. The essential oil of the leaves presented similar larvicidal toxicity with the cold pressed oil of the rind (LC_50_=68 mg/L).

## Introduction

The essential oil contained in the rind of the bergamot fruit (*Citrus aurantium subsp. bergamia,* syn. *Citrus bergamia* Risso et Poiteau) is a high value product with many applications in the perfume industry and cosmetology. It is also used in the food, beverage and confectionary industries as a flavoring for liqueurs, teas, candies and soft drinks. To date the highest quality bergamot oil is produced in the Ionian coast of Calabria (Italy) and has been well studied [1,2]. The plant is also cultivated in Ivory Coast, Brazil, Argentina and Turkey [3,4]. It is noteworthy that although the Ionian coasts are considered as the best region in the world for the cultivation of bergamot, in Greece the bergamot tree had never been cultivated for the production of essential oil. The bergamot trees studied herein were cultivated on Kefalonia Island (Vlachata region) at the same latitude as Reggio Calabria (38^o^06΄ N). 

The mature fruit of the bergamot variety found on Kefalonia is yellow, with a 10-20 cm diameter, 300-650 g weight, a peel thickness of approx. 2 cm, flavedo 20% and albedo 40% of total fruit wet weight. These characteristics make the fruit different from other common varieties of bergamot and could be considered as important advantages for its use for the production of essential oil or for the preparation of spoon sweets. 

In the present work we studied the composition and the quality of the produced bergamot oil over a two years period and additionally we studied its larvicidal activity against an anthropophilic mosquito biotype.

Among all arthropods, mosquitoes remain a serious disease vector as well as an important pest to humans, causing nuisance and allergic responses and in many areas of the world the command considerable attention in terms of their management and control [5,6,7]. The mosquito *Cx. pipiens* (Diptera: Culicidae) is a widespread mosquito species not only in Greece but also in other countries. This mosquito spp. was responsible in 1999 for the appearance of West Nile (WN) virus in the Western Hemisphere and also for a large outbreak in Southern Russia [8,9,10,11].

## Results and Discussion

Although the morphology of the fruit is different than that of the most common bergamot varieties, the composition of the obtained oil clearly shows a similarity with the oil obtained from Calabrian bergamots. In the present study, it was found that the main constituent of the cold pressed essential oil of the fruit rind was (–)-linalyl acetate with >99.9% optical purity, which is responsible for the characteristic odour of the bergamot fruit [4]. Other important constituents were (–)-linalool, (+)-limonene and *γ-*terpinene (Table 1).

**Table 1:** Composition of cold pressed oil of bergamot fruit harvested on 15^th^ of January showing the best quality values. The minimum and maximum percentage of each component has been recorded over a two year period. For comparison, the composition of the 15 January sample by atmospheric hydrodistillation is also presented as well as the min and max percentages of the main components of cold pressed Calabrian bergamot oil [3].

		Cold pressed			Distilled	Calabrian bergamot oil^h^
		15 January	min	max		min-max
	Compounds	%	%	%	%	%
1	α-Thujene	0.23	0.15	0.29	0.29	0.19-0.49
2	α-Pinene	0.13^a^	0.00	0.79	0.88	0.73-1.84
3	β-Pinene	0.23^b^	0.08	0.69	0.78	5.15-12.08
4	Myrcene	0.58	0.26	0.77	1.33	0.65-1.57
5	α-Phellandrene	0.01	0.01	0.01	0.05	0.02-0.06
6	α-Terpinene	0.16	0.00	0.23	0.30	0.08-0.28
7	Limonene	25.58^c^	10.54	34.88	31.66	25.62-53.19
8	*cis-*Ocimene	0.20	0.07	0.27	0.50	0.02-0.07
9	*trans*-β-Ocimene	0.24	0.12	0.24	0.79	0.10-0.36
10	γ-Terpinene	10.04	4.28	10.26	10.32	5.73-11.38
11	*cis*-Sabinene hydrate	0.10	0.05	0.10	-	0.02-0.06
12	Terpinolene	0.60	0.28	0.57	0.75	0.21-0.48
13	Linalool	15.33^d^	14.50	20.18	31.76^g^	1.75-20.26
14	Terpinen-4-ol	0.09	0.01	0.09	0.19	0.01-0.04
15	α-Terpineol	0.26^e^	0.01	0.33	3.85	0.03-0.10
16	Nerol	0.01	0.01	0.01	0.46	0.01-0.11
17	Neral	0.35	0.00	0.46	0.15	0.12-0.34
18	Linalyl acetate	40.51^f^	30.33	40.51	10.72	15.61-40.37
19	Geranial	0.48	0.01	0.83	0.11	0.25-0.49
20	Linalyl propionate	0.16	0.01	0.17	0.05	0.01-0.07
21	α-Terpinenyl acetate	0.35	0.01	0.35	0.00	0.09-0.26
22	Neryl acetate	0.00	0.01	0.07	0.70	0.14-0.67
23	Geranyl acetate	0.00	0.01	0.21	1.33	0.17-0.80
24	*trans*-Caryophyllene	0.34	0.11	0.59	0.20	0.22-0.52
25	α-Bergamotene	0.63	0.21	0.87	0.20	0.21-0.44
26	Humulene	0.00	0.00	0.04	0.00	0.02-0.04
27	Valencene	0.2	0.00	0.63	0.08	-
28	α-Bisabolene	0.08	0.00	0.12	0.00	-
29	β-Bisabolene	0.77	0.00	1.50	0.26	0.30-0.65
30	α-Bisabolol	0.00	0.00	0.10	0.00	0.01-0.03
	Linalool+linalyl acetate	55.84	44.5	55.8	42.48	
	Linalool/linalyl acetate	0.38	0.38	0.59	2.96	

^a^ (-)-α-Pinene: (+)-α-Pinene= 54:46, ^b^ (-)-β-Pinene: (+)-β-Pinene= 92:8, ^c^ (-)-Limonene: (+)-Limonene= 2:98, ^d^ (-)-Linalool: (+)-Linalool= 99.7:0.3, ^e^ (+)-α-Terpineol: (-)-α-Terpineol= 50:50, ^f^ (-)-Linalyl acetate: (+)-Linalyl acetate= 99.9: 0.1, ^g^ (-)-Linalool: (+)-Linalool= 90:10, ^h^ only common components or components with percentage >0.1% are presented

**Figure 1:**
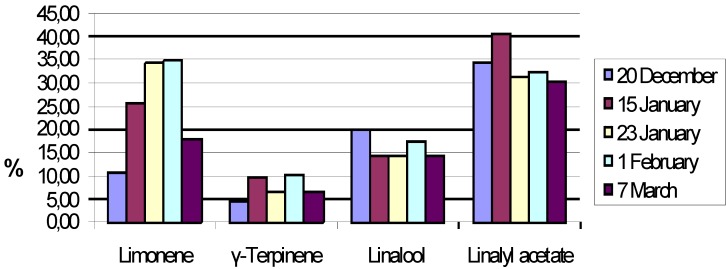
Variation of the main components concentration depending on harvesting time.

All the main constituents showed significant variations depending on the harvesting time (Figure 1). It should be noted that the main difference between Kefalonian bergamot oil and Calabrian or Turkish bergamot oil is the up to 10-fold reduced concentration of β-pinene/sabinene [1,2,3,4].

One important index for the characterization of the high quality bergamot oil is the linalool/linalyl acetate ratio, which for the Calabrian bergamot oil has been reported to be approximately 0.3 [1]. The performed analysis showed that the best value of this specific index (0.38) was achieved in bergamots harvested in mid January. Another characteristic index is the sum of linalool+linalyl acetate, which for the same harvest time (mid January) showed the maximum value (55.8%). In the bergamot oil of Calabria the corresponding index is reported to be approximately 50% [2]. 

Concerning the essential oil of the leaves it should be noted that it could be used as an alternative cheap source of bergamot-like oil. It is characterized by high linalool (34.6%) and linalyl acetate (29.8%) content and a low concentration of limonene (0.65%). Additionally neryl acetate (4.9%) and geranyl acetate (9.4%) are noticeably increased (Table 2).

**Figure 2:**
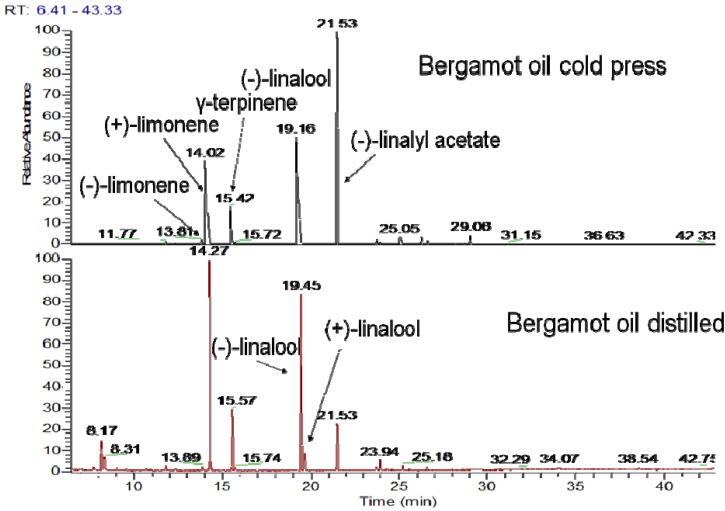
Comparison of bergamot oil obtained from the same raw plant material either by cold pressing or by hydrodistillation using GC-MS analysis with enantiomeric column b-DEX sm.

Concerning the fruit oil obtained by hydrodistillation it is noteworthy that its quality is lower due to the decomposition of linalyl acetate and the isomerization of linalool, as previously reported [4]. Comparison between the same sample treated by cold press or distillation reveals that the percentage of linalyl acetate falls from 40.5% to only 10.7% in the distilled oil (Table 1). Additionally, the thermal treatment for the distillation procedure leads to partial isomerization of the linalool, which can be confirmed by the presence of (+)-linalool (Figure 2).

**Table 2:** Composition of distilled essential oil from bergamot leaves.

Compound	%
β-Pinene	0.05
Myrcene	1.83
Limonene	0.65
*cis-O*cimene	1.13
*trans*-β-Ocimene	2.44
γ-Terpinene	0.88
Terpinolene	0.68
Linalool	34.62
Terpinen-4-ol	0.07
α-Terpineol	6.95
Decanal	0.1
Nerol	1.85
Neral	0.15
Linalyl acetate	29.8
Geranial	0.73
Linalyl propionate	0.38
Neryl acetate	4.85
Geranyl acetate	9.44
trans-Caryophyllene	0.48
α-Humulene	0.05
Caryophyllene oxide	0.06
Nonadecane	0.09
Total	99.31

### Larvicidal activity

The larvicidal effects of the essential oils against *Cx. pipiens* L. 3^rd^-4^th^ instar larvae are summarized in Table 3. To our knowledge, there is limited information on the larvicidal activity of *Citrus* sp essential oils against this genus of mosquito and no reports on comparative larvicidal detection for essential oils obtained by different procedures.

**Table 3:** LC_50_ and LC_90_ values for the bergamot fruit and leaves essential oil against *Cx. pipiens* biotype *molestus*; two different procedures for bergamot essential oil were tested (see text). Total mortality after 48 h was taken into consideration.

Tested material	LC_50_ (mg/L) (95% c.l.)^a^	LC_90_ (95% c.l.)^a^	Slope (±SE)
Essential oil type			
Bergamot (cold pressed)	58.73(54.75-62.87)	99.22(89.38 -114.94)	5.62±0.58
Bergamot (distillation)	106.6(96.7-126.24)	186.66(149.5-289.7)	5.26±0.91
Leaves	68.53(54.69-83.91)	151.6(116.97-245.6)	3.72±0.35^b^
Compounds			
(±)-Linalyl acetate	23.14(19.17-26,83)	41.78(35.09-56.09)	4.99±0.50
(±)-Linalool	> 200	––	––
(–)-Linalool	169.1(159.06-181.38)	268.54(234.54-347.75)	6.38±1.08

^a^ LC values are expressed in mg/L and they are considered significantly different when 95% c.l. fail to overlap; ^b^ Since goodness–of–fit test is significant (P<0.15), a heterogeneity factor is used in the calculation of confidence limits (c.l.)

Specifically, essential oil from lemon (*C. limon*) after 1-h contact with third-instar larvae of *Cx*. *quinquefasciatus* displayed a LC_50_ value near 50 ppm [12] and 80 ppm, when *Cx. pipiens* was exposed for 24-h to lemon peel crushed in water [13]. On the other hand, essential oil from *C. bergamia* exhibited 100% mortality against *Cx*. *p*. *pallens* at a dose of 25 ppm [14]. In the current project the essential oil obtained by cold pressing showed an LC_50_ value of 58.73 mg/L, while the LC_50_ value of the corresponding essential oil obtained by hydrostillation was 106.6 mg/L. The differentiation in LC_50_ values is due to the non similar tested species, the obtained essential oils and also to the tested method. 

In our project, the activity was found to be correlated primarily with the percentage of linalyl acetate and secondarily with some *p*-menthane type molecules. In details, the samples obtained by the first method (cold pressing) were rich (more than 75%) in limonene, *γ-*terpinene and linalyl acetate and showed the highest larvicidal toxicity. The LC_50_ values for *R-* and *S*-limonene are 43 to 53 mg/L, respectively, and *γ-*terpinene was found to be the most toxic *p*-menthane type substance, displaying an LC_50_ value of 10.4 mg/L [15]. Linalyl acetate and linalool are the main constituents of the bergamot fruit and leaves essential oils and their ratio or sum are two characteristic indexes for high quality oils. For larvicidal bioassays only racemic linalyl acetate seems to exhibit a significant toxic effect (LC_50_ value = 23.14 mg/L), while racemic linalool found to have no toxic effect against *Cx. pipiens* (LC_50_ value > 200 mg/L) (Table 3). Moreover, the toxicity pattern for racemic and (–) linalool was dissimilar and the LC_50_ value for the (–) form revealed little difference in toxicity from the racemic form. 

Among all substances that are greater than 10% in the tested essential oils, linalyl acetate and *γ-*terpinene are the most toxic. The activity found to be correlated with the percentage of aforementioned substances since the samples obtained by the second method had lower concentration and showed reduced larvicidal toxicity. Linalyl acetate was tested for the first time against *Culex* species and the results revealed a very good toxicity level, compared to other already known *p*-menthane type molecules. 

Finally, the essential oil obtained from the leaves presented similar larvicidal toxicity to the oil obtained by cold pressing of the rind (LC_50_=68 mg/L), even though these are differentiated in the identified constituents. Although the essential oil from leaves is rich in linalool and poor in *γ-*terpinene the presence of linalyl, geranyl and neryl acetate is more than 44%. All these esters are toxic and their LC_50_ value is near 25 mg/L (Michaelakis, unpublished data). This is an important finding since the leaves are a very cheap and more easily processed raw material in comparison with fruit.

According to the Annex I of the Directive 98/8/EC European Union some conventional larvicidals (e.g. organophosphates) are forbidden and therefore, there is an urgent need to develop new environmentally safe alternatives. From these standpoints plant-derived substances have received significant scientific attention as a potential source for the identification of novel natural mosquito control products [15,16,17,18,19,20,21,22]. 

## Conclusions

The high quality of the Kefalonian bergamot oil confirmed the observation that the Ionian coasts is one of the most appropriate regions in the world for the cultivation of bergamot tree. The present work offers important supporting data for the exploitation of Kefalonian bergamot as a new agroindustrial product in Greece and additionally as an interesting agent against mosquito larvae. 

## Experimental

### Plant material

Yellow fruits were collected between December and March for a two year period (2007-2008). Leaves were collected in one point time (15^th^ of January). The plant material was submitted to oil extraction within 24 h after its collection. Voucher specimens are deposited in the herbarium of the Laboratory of Pharmacognosy, University of Athens, Greece.

### Essential oil obtainment

The rind of the fruit (each sample 3 Kg) was separated manually and the essential oil was obtained either by cold pressing or by hydrodistillation for 3 hours in a Clevenger apparatus. The maximum yield calculated on wet weight of fresh rinds was 1.8%. The leaves (4 Kg) were submitted to hydrodistillation for 3 hours using a semi-industrial distiller affording 0.55% of yellow oil. The obtained oil were dried over anhydrous sodium sulphate and stored at 4^o^C until analysis. 

### Chemical analysis

The oils were first analyzed by GC-FID on a Perkin-Elmer Clarus 500 gas chromatograph, fitted with a HP 5MS 30 m x 0.25 mm, 0.25 μm film thickness capillary column. The column temperature was programmed from 60 °C to 280 °C at a rate of 3 °C/min. The injector and detector temperatures were programmed at 230 °C and 300 °C, respectively. Helium was used as the carrier gas at a flow rate 0.8 mL/min. The GC-MS analyses were carried out using a Hewlett Packard 6890-5973 GC-MS system operating on EI mode (equipped with a HP 5MS 30 m x 0.25 mm, 0.25 μm film thickness capillary column). He (0.8 mL/min) was used as carrier gas. The initial temperature of the column was 60 °C and then it was heated to 280 °C at a rate of of 3 °C /min. Chiral GC-MS was carried out on a Finnigan GCQ *Plus* mass spectrometer operating in EI mode equipped with a 30 m x 0.25 mm i.d.; 0.25 mm β-DEX™ (RESTEK) capillary column; temperature program: 60 °C to 220 °C at a rate of 4 °C/min; injection temperature 200 °C, He (1 mL/min). The identification of the compounds was based on comparison of their retention indices (RI), obtained using *n*-alkanes (C_9_-C_25_), and on comparison of their EI-mass spectra with the NIST/NBS, Wiley library spectra and literature [23]. The enantiomeric identification of the constituents was based on the co-injection with commercial or previously isolated standards as well as comparison with the corresponding mass spectra. The qualitative and quantitative composition of the essential oils is presented in Table 1 and Table 2.

### Mosquito Rearing

Mosquito larvae were collected from a colony that has been maintained in the laboratory of Benaki Phytopathological Institute, Kifissia, Greece for more than 20 years. Adults were kept in wooden framed cages (33x33x33 cm) with a 32x32 mesh at 25±2 °C, 80±2% relative humidity and photoperiod of 14:10 (L:D) h. Cotton wicks saturated with 10% sucrose solution were used as food source for the mosquitoes. Females laid eggs in round, plastic containers (10 cm diameter x 5 cm depth) filled with 150 mL of tap water. Egg rafts were removed daily and placed in cylindrical enamel pans (with diameter of 35 cm and 10 cm deep), in order to hatch. Larvae were reared under the above mentioned conditions of temperature and light and were fed daily with baby fish food (TetraMin, Baby Fish Food) at a concentration of 0.25 g/L of water until pupation. Pupae were then collected and introduced into the adult rearing cages.

### Larvicidal Bioassays

Stock solutions of each essential oil were prepared in ethanol with a concenration of 10% w/v. A series of aqueous solution with different concentration of essential oils, expressed as mg/L, was made and tested under laboratory conditions. The larval mortality bioassays were carried out according to the test method of larval susceptibility as suggested by the World Health Organization [24]. Twenty larvae were placed in glass beaker with 250 mL of aqueous suspension of essential oils at various concentrations. Four replicates were made per concentration and a control treatment with tap water was included in each bioassay. Beakers with larvae were placed at 25±2 °C, 80±2% relative humidity and photoperiod of 14:10 (L:D) h. The percentage of dead larvae was estimated and data were further subjected to Probit analysis (SPSS 11.0).

### Statistical analysis

Larvicidal effects were recorded 24 and 48 h after treatment. For the mortality caused after 48 h exposure to essential oil (total mortality, mg/L concentration in water) data were tested with Probit analysis (SPSS 11.0). Data obtained from each dose–larvicidal bioassay (total mortality, mg/L concentration in water) were subjected to probit analysis in which probit–transformed mortality was regressed against log_10_–transformed dose; LC_50_, LC_90_ values, and slopes were generated (SPSS 11.0). Four samples were used in each experiment (n = 4).

## Supplementary Files

Supplementary File 1
