# Correction: Eleni, M. *et al*. High Quality Bergamot Oil from Greece: Chemical Analysis Using Chiral Gas Chromatography and Larvicidal Activity against the West Nile Virus Vector. *Molecules* 2009*, 14(2),* 839-849

**DOI:** 10.3390/molecules14051948

**Published:** 2009-05-25

**Authors:** Eleni Melliou, Antonios Michaelakis, George Koliopoulos, Alexios-Leandros Skaltsounis, Prokopios Magiatis

**Affiliations:** 1 Department of Pharmacognosy and Natural Products Chemistry, Faculty of Pharmacy, University of Athens, Panepistimiopolis-Zografou, Athens 15771, Greece; E-mails: emelliou@pharm.uoa.gr (E.M), skaltsounis@pharm.uoa.gr (A-L.S.); 2 Laboratory of Agricultural Entomology, Department of Entomology and Agricultural Zoology, Benaki Phytopathological Institute, 8 S. Delta Str. 14561 Kifissia Athens, Greece; E-mail: a.michaelakis@bpi.gr (A.M.); 3 Laboratory of Insecticides of Public Health Importance, Benaki Phytopathological Institute, 8 S. Delta str. 14561 Kifissia Athens, Greece; E-mail: g.koliopoulos@bpi.gr (G.K.)

In the original published version of this paper [1], the given names and surnames of the coauthors were accidently inverted, and should be as indicated below: 

**Eleni** **Melliou ^1^, Antonios** **Michaelakis ^2^, George** **Koliopoulos ^3^, Alexios-Leandros** **Skaltsounis ^1^ and Prokopios** Magiatis ^1,^*

^1^ Department of Pharmacognosy and Natural Products Chemistry, Faculty of Pharmacy, University of Athens, Panepistimiopolis-Zografou, Athens 15771, Greece; E-mails: emelliou@pharm.uoa.gr (E.M), skaltsounis@pharm.uoa.gr (A-L.S.)

^2^ Laboratory of Agricultural Entomology, Department of Entomology and Agricultural Zoology, Benaki Phytopathological Institute, 8 S. Delta Str. 14561 Kifissia Athens, Greece; E-mail: a.michaelakis@bpi.gr (A.M.)

^3^ Laboratory of Insecticides of Public Health Importance, Benaki Phytopathological Institute, 8 S. Delta str. 14561 Kifissia Athens, Greece; E-mail: g.koliopoulos@bpi.gr (G.K.)
