# Supplementary material for: High Quality Bergamot Oil from Greece: Chemical Analysis Using Chiral Gas Chromatography and Larvicidal Activity against the West Nile Virus Vector
Source: Molecules. 2009 Feb 18;14(2):839–49. doi: 10.3390/molecules14020839 (PMC6253838; doi:10.3390/molecules14020839)
Supplement: Supplementary File 1 [file molecules-14-00839-s001.pdf]

Molecules **2009**, *14*, 1948-1949; doi:10.3390/molecules14051948

OPEN ACCESS

molecules

ISSN 1420-3049

www.mdpi.com/journal/molecules

Correction

Correction: Eleni, M. *et al*. High Quality Bergamot Oil from Greece: Chemical Analysis Using Chiral Gas Chromatography and Larvicidal Activity against the West Nile Virus Vector. *Molecules* 2009, *14(2)*, 839-849

Eleni Melliou [1], Antonios Michaelakis [2], George Koliopoulos [3], Alexios-Leandros Skaltsounis [1] and Prokopios Magiatis [1,*]

[1] Department of Pharmacognosy and Natural Products Chemistry, Faculty of Pharmacy, University of Athens, Panepistimiopolis-Zografou, Athens 15771, Greece; E-mails: emelliou@pharm.uoa.gr (E.M), skaltsounis@pharm.uoa.gr (A-L.S.)

[2] Laboratory of Agricultural Entomology, Department of Entomology and Agricultural Zoology, Benaki Phytopathological Institute, 8 S. Delta Str. 14561 Kifissia Athens, Greece; E-mail: a.michaelakis@bpi.gr (A.M.)

[3] Laboratory of Insecticides of Public Health Importance, Benaki Phytopathological Institute, 8 S. Delta str. 14561 Kifissia Athens, Greece; E-mail: g.koliopoulos@bpi.gr (G.K.)

* Author to whom correspondence should be addressed; E-mail: magiatis@pharm.uoa.gr

Received: 14 May 2009/ Published: 25 May 2009

In the original published version of this paper [1], the given names and surnames of the coauthors were accidently inverted, and should be as indicated below:

Eleni Melliou [1], Antonios Michaelakis [2], George Koliopoulos [3], Alexios-Leandros Skaltsounis [1] and Prokopios Magiatis [1,*]

[1] Department of Pharmacognosy and Natural Products Chemistry, Faculty of Pharmacy, University of Athens, Panepistimiopolis-Zografou, Athens 15771, Greece; E-mails: emelliou@pharm.uoa.gr (E.M), skaltsounis@pharm.uoa.gr (A-L.S.)

[2] Laboratory of Agricultural Entomology, Department of Entomology and Agricultural Zoology, Benaki Phytopathological Institute, 8 S. Delta Str. 14561 Kifissia Athens, Greece; E-mail: a.michaelakis@bpi.gr (A.M.)

[3] Laboratory of Insecticides of Public Health Importance, Benaki Phytopathological Institute, 8 S. Delta str. 14561 Kifissia Athens, Greece; E-mail: g.koliopoulos@bpi.gr (G.K.)

Reference

1. Eleni, M.; Antonios, M.; George, K.; Alexios-Leandros, S.; Prokopios, M. High Quality Bergamot Oil from Greece: Chemical Analysis Using Chiral Gas Chromatography and Larvicidal Activity against the West Nile Virus Vector. *Molecules* **2009**, *14*, 839-849.

© 2009 by the authors; licensee Molecular Diversity Preservation International, Basel, Switzerland. This article is an open-access article distributed under the terms and conditions of the Creative Commons Attribution license (http://creativecommons.org/licenses/by/3.0/).
